# Quantifying the potential for bursting bubbles to damage suspended cells

**DOI:** 10.1038/s41598-017-14531-5

**Published:** 2017-11-08

**Authors:** Peter L. L. Walls, Oliver McRae, Venkatesh Natarajan, Chris Johnson, Chris Antoniou, James C. Bird

**Affiliations:** 1 Department of Mechanical Engineering, Boston University, Boston, MA 02215 USA; 2 Global Processing Engineering, Biogen, Cambridge, MA 02142 USA

## Abstract

Bubbles that rise to the surface of a cell suspension can damage cells when they pop. This phenomenon is particularly problematic in the biotechnology industry, as production scale bioreactors require continuous injection of oxygen bubbles to maintain cell growth. Previous studies have linked cell damage to high energy dissipation rates (EDR) and have predicted that for small bubbles the EDR could exceed values that would kill many cells used in bioreactors, including Chinese Hamster Ovary (CHO) cells. However, it’s unclear how many cells would be damaged by a particular bursting bubble, or more precisely how much volume around the bubble experiences these large energy dissipation rates. Here we quantify these volumes using numerical simulations and demonstrate that even though the volume exceeding a particular EDR increases with bubble size, on a volume-to-volume basis smaller bubbles have a more significant impact. We validate our model with high-speed experiments and present our results in a non-dimensionalized framework, enabling predictions for a variety of liquids and bubble sizes. The results are not restricted to bubbles in bioreactors and may be relevant to a variety of applications ranging from fermentation processes to characterizing the stress levels experienced by microorganisms within the sea surface microlayer.

## Introduction

**Figure 1:**
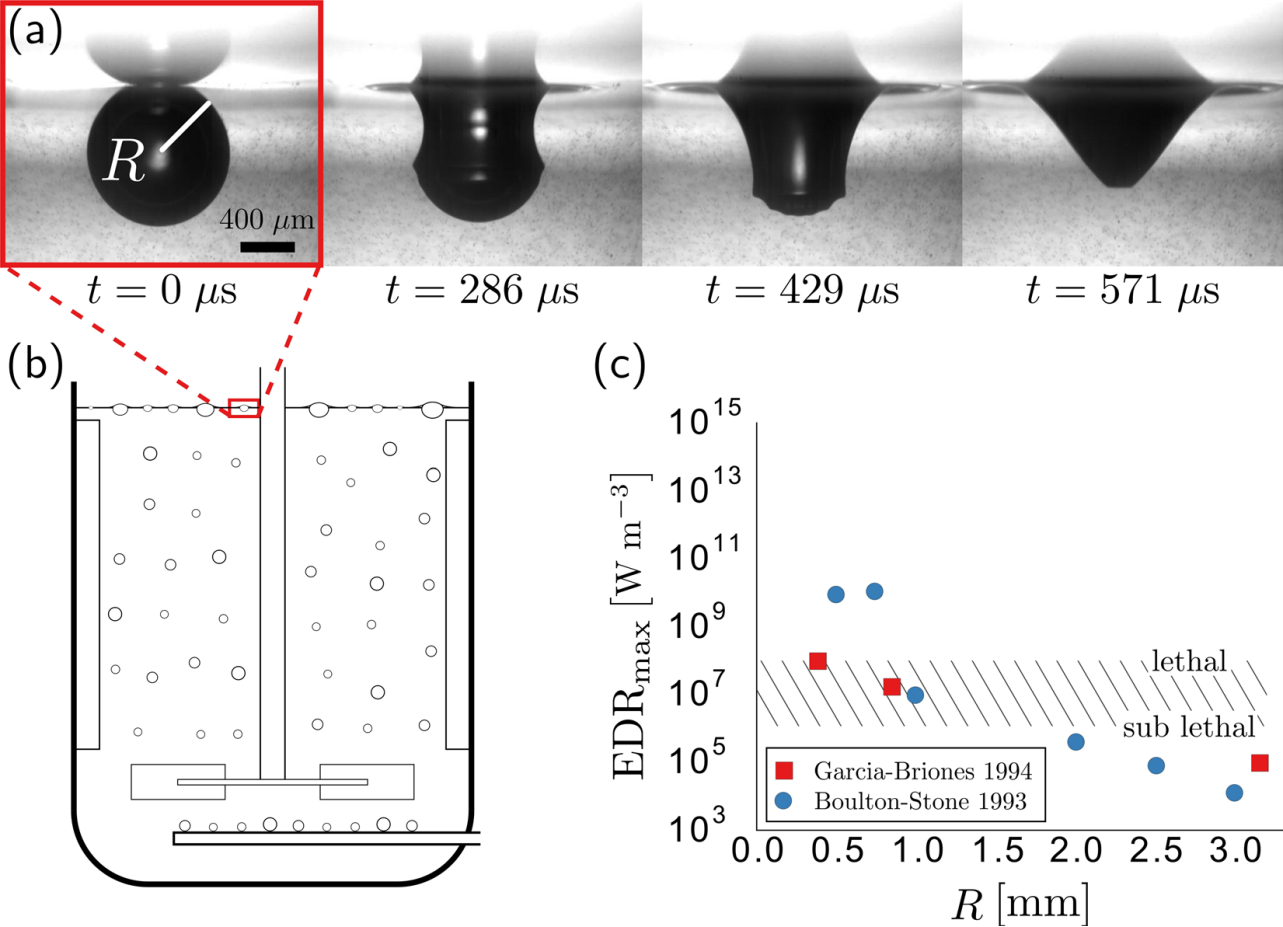
The spontaneous rupture of bubbles is known to cause damage to cells grown in bioreactors. (**a**) Immediately after rupture, capillary waves travel down the sides of the bubble in approximately *t* = 571 *μ*s for a bubble of radius *R* = 0.5 mm. (**b**) Bubbles are commonly injected directly near the base of the bioreactors to continually oxygenate the cells and remove excess carbon dioxide. (**c**) Past numerical studies have concluded that the smallest bubbles tend to produce the highest levels of overall maximum energy dissipation rate (EDR_max_). Mammalian CHO cells exposed to EDR levels exceeding values ranging from 10^6^ to 10^8^ W/m^3^ (hatched region) exhibit a lethal response (necrosis, including lactate dehydrogenase release).

The biotechnology industry has long recognized the potential for excessive hydrodynamic stresses to kill and reduce the viability of animal cell cultures grown in suspension^1–8^. In addition, sub-lethal stresses have been shown to negatively impact a cell’s production of protein^4,9^. In sparged bioreactors, past studies have shown that the majority of damage to cells grown in suspension is caused by the high stresses originating from bubbles bursting at the free surface (Fig. 1a,b)^4,10–14^. Because of the damage caused by these rupturing bubbles, protective additives, such as Pluronic F-68, are often added to help mitigate damage by preventing bubble-cell attachment. However, there is evidence that cells are concentrated in the upper layer of a bioreactor, even if additives fully prevent bubble-cell attachment^15^. Although there is consensus that cells attached to the bubble’s interface will be damaged when a bubble ruptures, it is less clear how a nearby cell would be damaged.

The high stresses from a bursting bubble are a result of the rapid rearrangement of its interface. It takes less than a millisecond for the cavity of a bubble with an initial radius *R* ≈ 0.5 mm to return to equilibrium after spontaneously rupturing (Fig. 1a). Within this fraction of a millisecond, surface tension-driven capillary waves travel down the side of the cavity, collide at the bottom and produce upward and downward traveling jets^16^. Several studies have numerically simulated this rupture process^12,17–20^. In particular, two of these studies^12,17^ have been widely cited in the biotechnology literature because they report a maximum energy dissipation rate (EDR) associated with the bubble rupturing process. EDR is a scalar value that quantifies the rate of work done by the surrounding liquid on a fluid volume and has long been used as a measure to relate hydrodynamic flow stresses and cell damage^21^. For example, cell death for the commonly used Chinese Hamster Ovary (CHO) cell occurs when these cells are exposed to a single flow event with a characteristic EDR that exceeds values ranging between 10^6^ to 10^8^ W m^−3^ ^9^. A similar range of EDR is associated with cell death in several other suspended cell lines relevant to the biotechnology industry, including Hela S3 and Hybridoma cells^4^. Furthermore, when cells are exposed to repeated events, the EDR thresholds drops by nearly two orders of magnitude^8^.

In the context of bubble rupturing, both previous numerical studies predict an overall (temporal and spatial) maximum energy dissipation rate EDR_max_ that ranges from approximately 10^4^ to 10^10^ W m^−3^, well within the damaging range for these suspended cell lines (Fig. 1c). However, it is unclear why these two studies differ in their predicted maximum EDR. For example, the EDR_max_ reported for the smallest bubbles varies by more than two orders of magnitude (Fig. 1c)^12,17^. Additionally, it is unclear how large a volume surrounding the bubble might experience EDR levels close to the reported maximum. Finally, it is unclear how the reported values of EDR might change if the surrounding liquid were not pure water.

Here, we carryout a series of benchmarked simulations to model the flow associated with a rupturing bubble at a free surface. These simulations allow us to demonstrate why relying on a single value of EDR_max_ can be problematic. Instead, we propose reporting liquid volumes associated with given EDR_max_ thresholds. We calculate these volumes around a bursting bubble by incorporating particle tracking within our simulations. Finally, we generalize our results by recasting them in dimensionless variables. This approach allows our results to be applied more broadly, such as in the biotechnology industry, where the fluid properties of the cell growth media may differ from pure water.

## Computational Method

A volume-of-fluid method is used to compute the flow field around a rupturing bubble at the free surface. Specifically, we use the open-source flow solver Gerris, to solve the full axisymmetric Navier-Stokes equations. Gerris utilizes an adaptive grid refinement, allowing for nearly five orders of magnitude in spatial resolution^22,23^. This improved spatial resolution is especially important for modeling surface tension-driven flows as they tend to produce complex geometries spanning several orders of magnitude^23,24^. Furthermore, we assume both the liquid and gas phases are incompressible.

Similar to previous studies, we begin simulations with a single bubble resting at the free interface. Bubbles typically rest on the surface momentarily before rupturing, adopting an equilibrium shape based on a balance of the gravitational and surface tension forces. The resulting shape is unique and determined by the value of the dimensionless Bond number Bo ≡ *ρgR* ^2^/*γ*, where *ρ* is the liquid density, *g* is the acceleration due to gravity, and *γ* is the surface tension^25^. The bubble shape for small Bond numbers approximates a sphere that is mostly submerged (See Fig. 1a), whereas the shape for larger Bond numbers approaches that of a hemisphere that rests above the liquid surface. To simplify the geometry of our model, we remove the thin film of the spherical cap, leaving only the bubble cavity. This numerical approach has been shown to provide good agreement with experiments^19^ and is commonly done when simulating bubble rupture^12,17,18^. The removal of the spherical cap can also be justified by comparing the timescale of the film retraction $\sqrt{\rho {R}^{2}h/2\gamma }$ with the timescale of the bubble collapsing $\sqrt{\rho {R}^{3}/\gamma }$ resulting in $\sqrt{h/2R}$, where *h* is the thickness of the bubble cap at rupture. For example, the *h* = 0.1 *μ*m cap of a *R* = 500 *μ*m bubble retracts in a hundredth of the time the cavity takes to collapse and may safely be ignored. Once the geometry has been initialized, the simulation begins and the forces associated with surface tension rapidly rearrange the interface.

### Experimental Validation

**Figure 2:**
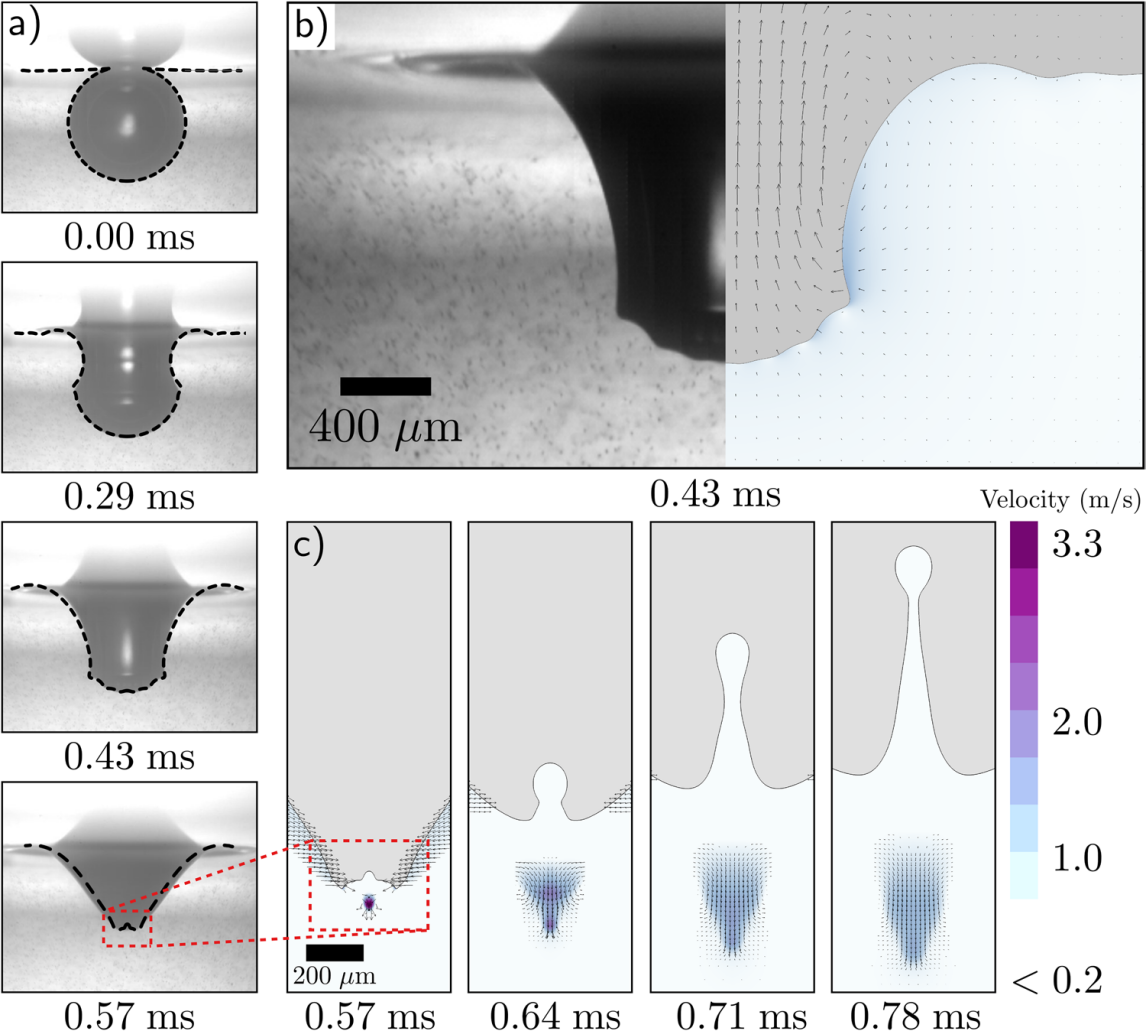
A direct comparison between our high-speed bubble bursting experiment and the corresponding simulation. (**a**) The interface predicted by the simulations is overlayed (black dashed line) onto the experimental profile from Fig. 1a. (**b**) A side-by-side comparison demonstrates that the fine features of the capillary waves immediately before colliding at the bottom of the bubble are well defined in the simulation result. (**c**) Upward and downward traveling jets are formed immediately after the capillary waves collide and focus the flow. Here the magnitude of the downward velocity is plotted.

To ensure that our simulations accurately capture the bubble rupture physics, we directly compare our numerical results with high speed images from a corresponding experiment. In the experiment, we inject an air bubble into a water bath that has been seeded with micron sized beads to help visualize the flow. The bubble establishes a nearly spherical shape before spontaneously rupturing (Fig. 2a). To compare the bubble rupture dynamics, we overlay our experimental result with the interface extracted from the numerical result (dashed line) at the shown times after rupture *t* (Fig. 2a). A side-by-side comparison at *t* = 430 *μ*s illustrates that the simulations capture the fine details of the capillary waves near the bottom of the bubble (Fig. 2b). Furthermore, comparison of the velocity magnitude of the downward jet with our particle tracking result also shows good agreement between the numerical and experimental result (Fig. 2c). Collectively, these results provide confidence that our simulations accurately capture the dynamics of bubble rupture, including the velocity field of the surrounding flow.

## Results and Discussion

### Revisiting Past Numerical Studies

A series of simulations are performed to investigate why the previous numerical studies of Boulton-Stone *et al*.^12^ and Garcia-Briones *et al*.^17^ disagree to such an extent on the reported values of EDR_max_ despite having a similar computational approach. To enable a direct comparison, we follow the methods of the previous two studies and record the absolute largest value of EDR in regions where the phase is wholly liquid, and therefore unquestionably off of the interface. The maximum energy dissipation EDR_max_ denotes the largest EDR value computed in the liquid over the entire duration of the simulation, from the initial bubble rupture until the flow returns to a near-quiescent state. A total of 27 simulations are carried out— three levels of refinement for each of the nine bubble sizes reported in the prior studies. Here, our base mesh resolution corresponds to 1024 cells per bubble radius *R*, or equivalently a mesh size of 0.38 *μ*m for the smallest bubble (*R* = 385 *μ*m) simulated. In addition to this base resolution, we compare the results with mesh resolutions of 2× and 4× the base resolution.

**Figure 3:**
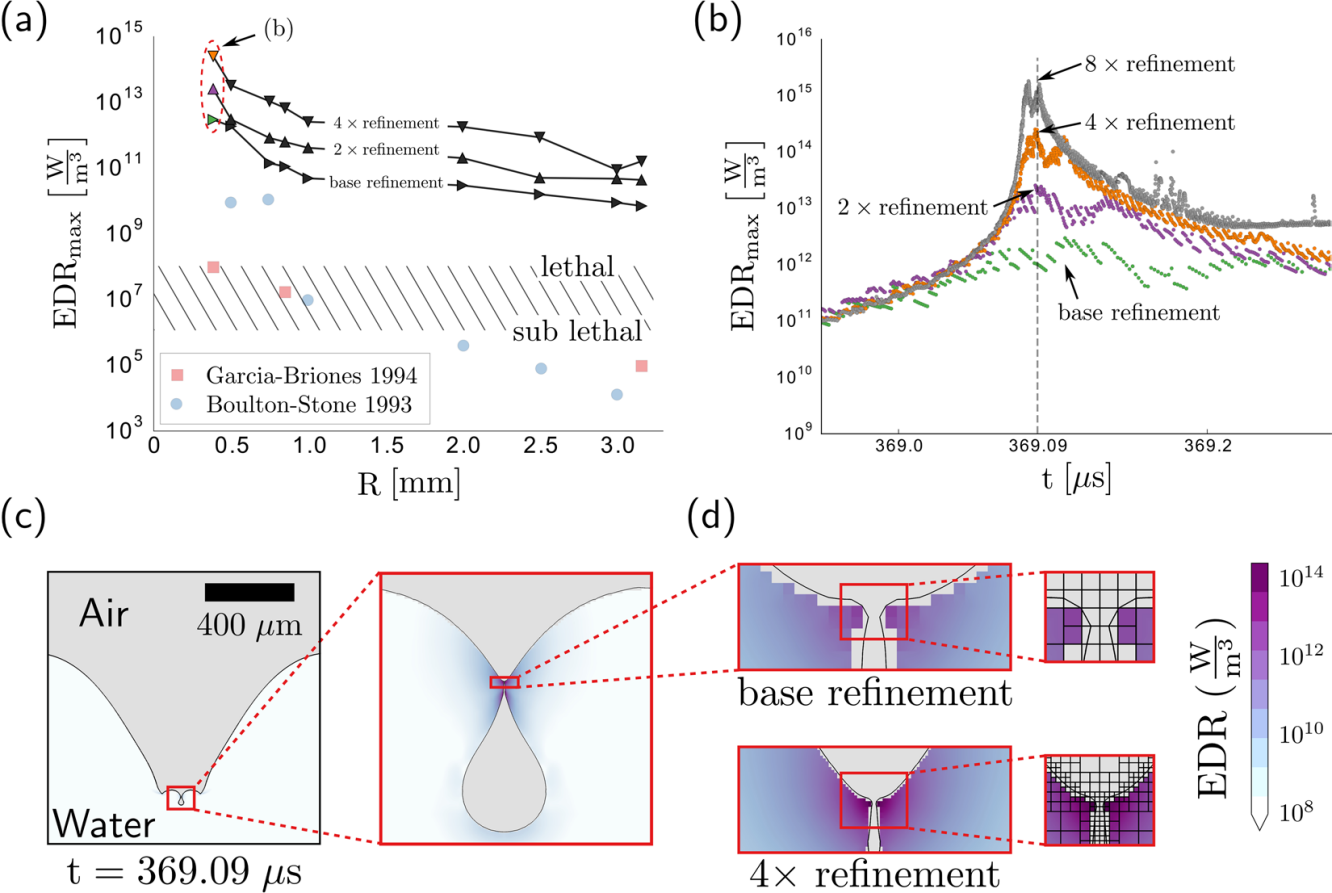
Compilation of individual bubble bursting simulations. (**a**) In contrast to previous numerical studies (red squares and blue circles), we find that all bubbles produce an overall maximum level of EDR exceeding the generally accepted lethal threshold for CHO cells (hatched region) and that this maximum depends on the level of mesh refinement. (**b**) The spatial maximum of the EDR varies with time *t* and reaches a temporal maximum at *t* ≈ 369 *μ*s for the *R* = 385 *μ*m air bubble cases circled in part (**a**). (**c**) The time at which the maximum EDR is reached, capillary waves have converged at the base of the bubble to create a region of high-curvature that pinches off to form a small bubble. (**d**) The local features and corresponding EDR_max_ depend on the level of mesh refinement, as might be expected from the singular dynamics associated with these capillary waves.

Figure 3a displays the calculated value of EDR_max_ for each of the bubble sizes and corresponding mesh resolution. One striking result is that the EDR_max_ values from our simulations are orders of magnitude higher than those reported in the previous works. Therefore, contrary to the implications of the earlier numerical studies, we conclude that all bubbles within the range probed would have regions lethal to CHO cells suspended in the flow. This result, that larger bubbles are also lethal, is consistent with experimental observations. For example, experiments carried out with 1.75 mm radius bubbles reported death rates exceeding 70% for SF-9 cells^26^, a suspension grown cell with a similar resilience to hydrodynamic stresses as the CHO cell^7^.

Further inspection of Fig. 3 reveals that the value of EDR_max_ does not appear to converge and continues to increase with increasing mesh refinement. In general, solution divergence is undesirable when performing numerical simulations. However, this particular divergence does not appear to be a flaw with the simulations, but rather a consequence of the fluid dynamics of the bubble rupture itself.

To illustrate why the EDR_max_ diverges with increasing mesh resolutions, we compare the three simulations for the *R* = 385 *μ*m case in further detail (circled in Fig. 3a). For each simulation, the value of EDR_max_ corresponds to an EDR at a particular time and location that is, by definition, larger than the EDR at all other times and locations. By measuring the spatial maximum of the EDR at each point in time *t*, we find that the temporal maximum for all three mesh resolutions occurs at approximately the same time (Fig. 3b). At approximately 369 *μ*s after the bubble ruptures, the spatial EDR maximum diverges sharply, with increased mesh refinement leading to the orders of magnitude higher values of EDR_max_ reported in Fig. 3a.

The source of the divergence can be identified by plotting the bubble interface shape and EDR at this critical time (Fig. 3c,d). Following rupture, capillary waves rapidly travel down the side of the submerged bubble (as illustrated in Fig. 2a). For the *R* = 385 *μ*m bubble, these waves reach the bottom at *t* ≈ 369 *μ*s, at which point they have highly curved the interface and created the conditions for an upward and downward jet (Fig. 3c). In some instances, the interface is curved enough to undergo a topological change and pinch-off to form a small bubble, a result that is observed in experiments^16^. This pinch-off event is a hallmark of a finite-time singularity, and leads certain parameters to approach infinity at the precise pinch-off time. Therefore extracting meaningful values of the EDR where and when the waves approach each other can prove problematic because, in the continuum limit, the relevant length and time scales can become arbitrarily small.

Numerically, increasing the level of mesh refinement near a finite-time singularity simply acts to reduce the smallest local length scale *δ* to the corresponding smallest mesh size (Fig. 3d). From this observation, we can estimate how much of an increase in EDR might result from doubling the mesh refinement. Because the energy dissipation rate is proportional to the square of the strain rate of the fluid (${\rm{EDR}}\propto {(\nabla {u}_{c})}^{2}\propto {t}_{c}^{-2}$) and the diverging velocity is based on a characteristic time $\sqrt{\rho {\delta }^{3}/\gamma }$, reducing the grid size *δ* by a factor of two should increase the EDR nearest the singularity by a factor:

1 $$\frac{{{\rm{EDR}}}_{2{\rm{x}}}}{{{\rm{EDR}}}_{{\rm{base}}}}\approx \frac{{({t}_{c}^{-2})}_{2{\rm{x}}}}{{({t}_{c}^{-2})}_{{\rm{base}}}}=\frac{\gamma /\rho \,{(\delta /2)}^{3}}{\gamma /\rho {\delta }^{3}}=8.$$

This simple scaling argument predicts that each time that the mesh resolution doubles, the EDR increases by approximately one order of magnitude. Indeed, the mesh resolution dependence illustrated in Fig. 3b supports this estimation.

We believe that mesh resolution dependence is the primary reason that past studies^12,17^ disagreed to such a large extent in their reported values of EDR_max_. Indeed, our computed values of EDR_max_ also disagreed with these past studies and indicated that large, as well as small, bubbles have the potential to damage cells. Yet, the amount of disagreement depends on the mesh resolution, with the increasing EDR values occurring over a diminishing volume. Therefore, we propose to move away from EDR_max_ as the main metric to quantify the potential damage caused by rupturing bubbles and adopt an approach that quantifies the volume of fluid that experiences, or exceeds, a given EDR value. This new metric requires that we track individual parcels of fluid to determine the EDR that cells within them would experience.

### Numerical Particle Tracking

Thus far, gravitational effects have been included in the analysis. In the remaining analysis, we consider small bubbles for which gravitational effects are neglected. This limit is helpful in two ways. First, removing a parameter (here the Bond number) simplifies the results and allows them to be applied to different liquids more easily. Second, by neglecting gravity, the initial shape of the bubble is spherical and does not vary with size, which simplifies the particle tracking between simulations. Even though the actual shape of the bubble will deviate from a fully submerged sphere, there is minimal deviation for bubbles with a Bond number less than 1, or equivalently water bubbles with radii less than *R* ≈ 2.5 mm. For the present study, we focus on bubbles ranging in size from *R* = 100 *μ*m to 1.5 mm, which are below this criterion.

**Figure 4:**
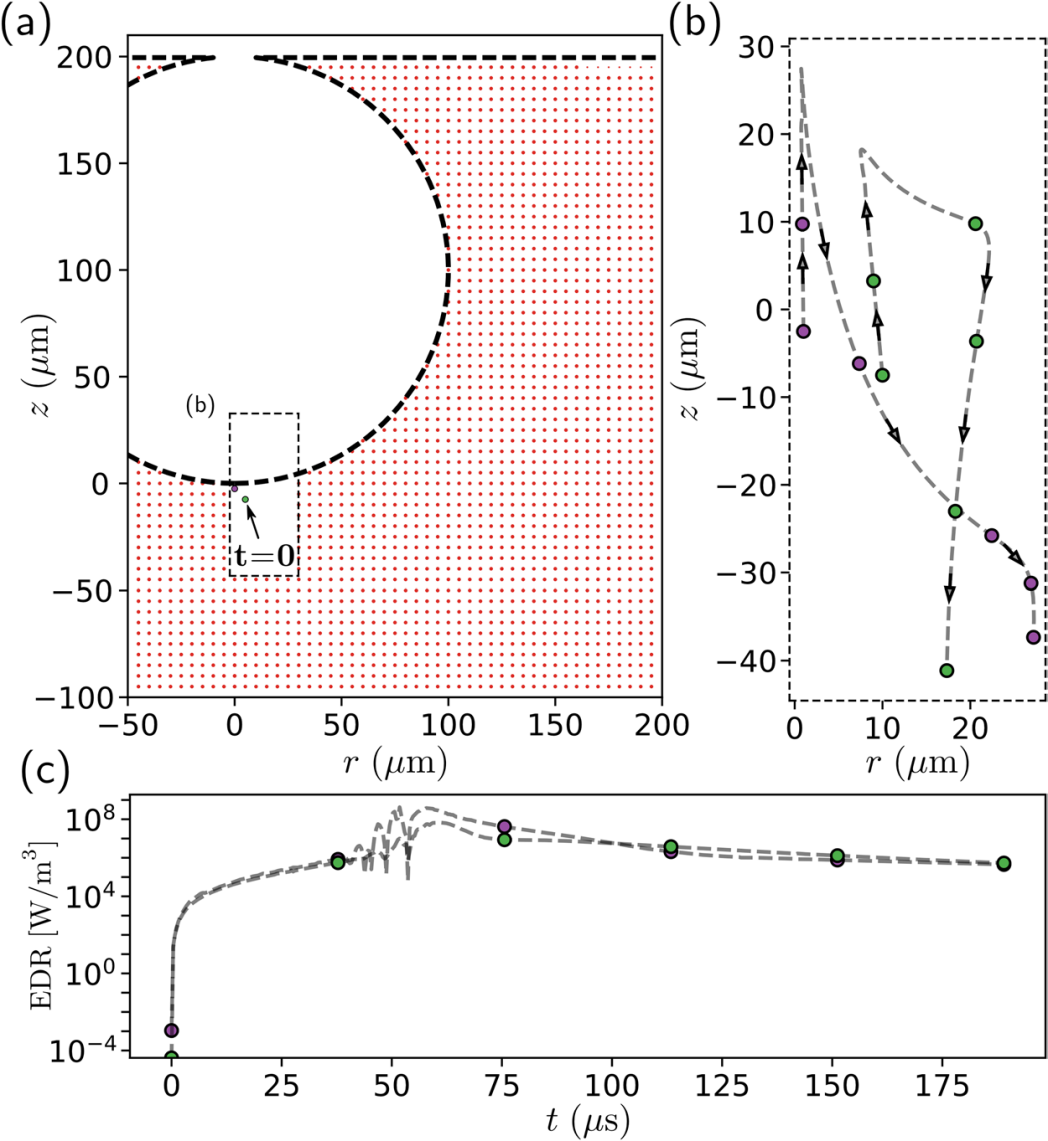
Overview of numerical tracking method. (**a**) At *t* = 0, the liquid phase of the simulation is seeded with a grid of numerical particles. (**b**) As the simulation progresses the position of each particle is tracked. As an example, we plot the trajectory of two particles that are initially pulled upwards before being propelled downwards in the liquid jet shown earlier in Fig. 2c. (**c**) Along with the position of each particle, the EDR experienced at each point is also recorded. We see that the particles experience EDR values ranging over several orders of magnitude.

To determine the EDR experienced by cells within a particular region, individual points are tagged and tracked during the bubble rupture simulation (Fig. 4). Prior to rupture, we consider a uniform grid of passive points, or particles, in the liquid phase surrounding the bubble, such as those illustrated by the red dots in Fig. 4a. The grid implemented in our simulations consists of nearly 500,000 particles to ensure sufficient spatial resolution. For the 100 *μ*m bubble shown in Fig. 4a this equates to a particle for every 0.5 *μ*m step in the axial and radial directions.

Once the bubble ruptures and the simulation begins, the position and corresponding EDR of each particle is recorded over time. To illustrate the utility of this approach, the paths of two particles are highlighted in Fig. 4. At the time of rupture, *t* = 0, both particles are positioned slightly below the bubble (Fig. 4a). As the simulation progresses, both particles are pulled upwards in the positive *z* direction before being propelled away from the bubble (Fig. 4b). As the particle begins to move, the EDR it experiences rapidly increases before reaching a maximum value approaching 10^9^ W m^−3^ (Fig. 4c). Here, the filled markers indicate equal time intervals of nearly 40 *μ*s along the particles’ trajectories and corresponding values of EDR. Figure 4c demonstrates that the maximum EDR experienced by the particles occurs at approximately *t* = 60 *μ*s. This time coincides with the particles rapidly changing direction (Fig. 4b) and is likely due to the downward jet (seen earlier in Fig. 2c). Once this method is applied to each particle in the grid, the maximum EDR experienced by a particle or cell around the bubble can be quantified based on its original position.

### Quantifying the Extent of Elevated EDR Levels

**Figure 5:**
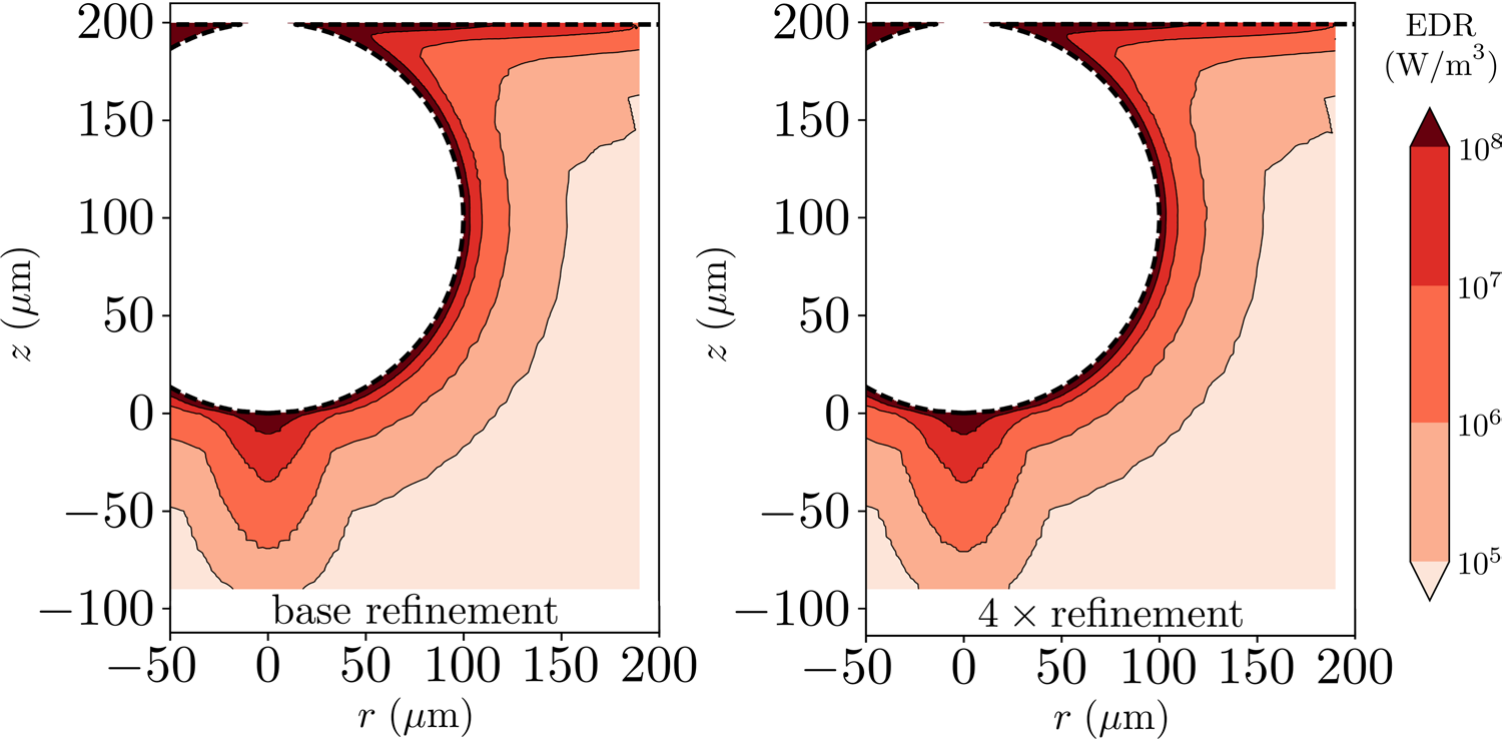
Maximum EDR experienced by a particle over the duration of the bubble rupture based on its initial position. We see that the region immediately adjacent to the bubble experiences EDR levels in excess of 10^8^ W m^−3^. The region of high EDR values extends further into the surroundings near the bottom of the bubble, demonstrating the influence of the downward jet. In contrast to the previous method of extracting a single maximum EDR value, increasing the mesh resolution by 4× does not alter the results.

As an alternative metric to an overall EDR_max_, we quantify the volume of the region surrounding the bubble that experiences an EDR level above a specified series of thresholds. Figure 5 depicts the initial position of each ‘particle’ of water surrounding a *R* = 100 *μ*m air bubble, color-coded with the corresponding maximum EDR experienced following rupture. This contour plot illustrates the regions most likely to cause damage to a nearby cell based on their resilience or chosen damage threshold. For example, our results indicate that cells initially positioned in the thin shell surrounding a 100 *μ*m bubble will experience energy dissipation rates in excess of 10^8^ W m^−3^, likely leading to cell death for CHO cells. This conclusion is in agreement with past experimental work demonstrating that a thin layer surrounding the bubble experiences lethal levels of stress^16,26^. Additionally, increasing the simulation mesh resolution by 4× from the base resolution does not influence the resulting contour plot (Fig. 5). Even though the large values of EDR_max_ shown in Fig. 3 are still present in our simulations, the value occurs over such a small amount of volume that its overall impact is negligible. Indeed, Fig. 5 illustrates not only that a cell’s initial distance from a bubble’s interface affects the EDR that it experiences, but also provides a quantitative spatial map. It is noteworthy that the contours of maximum EDR experienced are not concentric spheres; in particular, the contours below the bubble extend disproportionately into the liquid. This extended region highlights the impact of the downward jet and its focusing effect on the cells positioned directly below.

To calculate the volume of the liquid that experiences an EDR level greater than a specified threshold, we integrate the appropriate regions identified in Fig. 5. For example, when the specified threshold is 10^8^ W m^−3^, only the region contained within the thin shell nearest to the bubble is considered and yields a total volume of V = 0.64 nL. Although this volume may appear small in absolute terms, in terms of the original bubble volume V_b_ = 4.2 nL, the relative volume is over a tenth (V/V_b_ = 0.15). If the threshold is an order of magnitude lower at 10^7^ W m^−3^, then the two closest regions are integrated and yield V = 2.1 nL, or in relative terms, nearly half the original bubble volume (V/V_b_ = 0.49).

**Figure 6:**
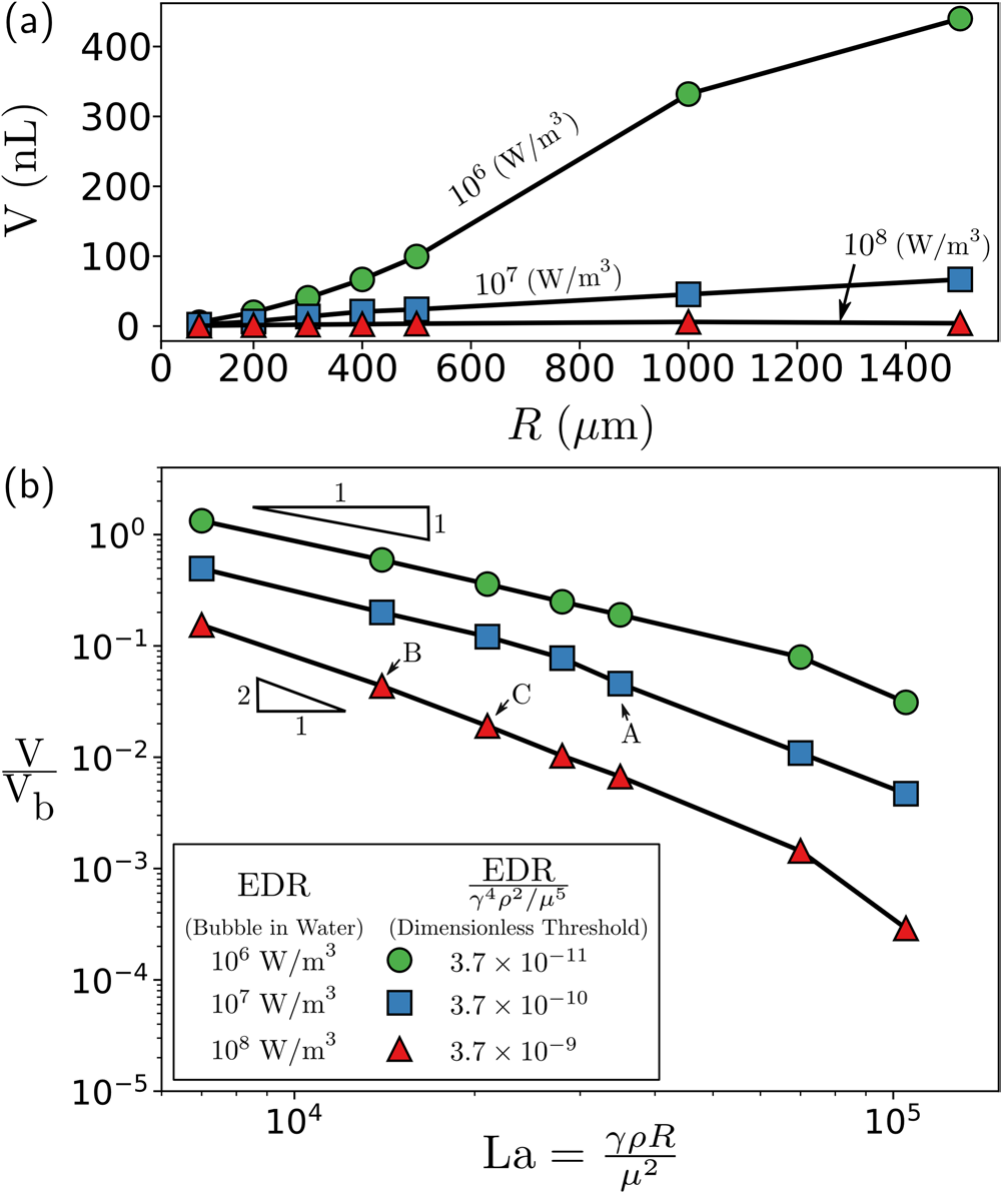
Volume of the liquid surrounding a bubble experiencing a level of EDR above the specified threshold. (**a**) The amount of volume increases for increasing bubble size for air bubbles rupturing in water. (**b**) However, in the dimensionless framework, the amount of volume experiencing an EDR above the specified threshold, in terms of the initial bubble size, decreases with increasing bubble size or dimensionless radius *γρR*/*μ* ^2^.

To extend these results, we apply these procedures to additional spherical bubbles with sizes that range from *R* = 200 *μ*m to *R* = 1500 *μ*m. Figure 6a illustrates that larger bubbles affect larger volume regions of the surrounding liquid. However, since aeration processes are typically controlled by injecting a pre-determined amount of oxygen per unit time, it is perhaps more useful to recast all of the results in terms of the volume fraction V/V_b_. Dimensional analysis suggests that in the absence of gravity, the bubble dynamics depend on a single dimensionless parameter that relates the bubble size to a inertial-capillary-viscous length scale *R*_v_ = *μ* ^2^/*γρ*—often written as a Laplace number La ≡ *γρR*/*μ* ^2^. Replotting our data for a bubble bursting in water in terms of V/V_b_ and La reveals that, although larger bubbles affect a larger absolute volume than smaller bubbles, the smaller bubbles affect a larger volume relative to their size (Fig. 6b). Finally, we rescale each of the EDR thresholds in Fig. 6b by a characteristic value that depends solely on the liquid properties EDR_c_ = *γ* ^4^ *ρ* ^2^/*μ* ^5^ (right side of legend in Fig. 6b). This approach enables predictions of the volume experiencing an EDR value above a specified threshold for a range of bubble sizes and liquid properties, such as viscosity and surface tension.

We illustrate the power of the dimensionless framework by directly comparing three cases in which the bubble size is fixed to *R* = 500 *μ*m and the threshold is fixed to EDR = 10^7^ W m^−3^. We begin with an air bubble bursting in water, which corresponds to a Laplace number La = 3.5 × 10^4^, as indicated by point A in Fig. 6b. For this chosen EDR threshold and bubble configuration, approximately V/V_b_ = 4.6% of the surrounding liquid volume exceeds damaging stress levels over the course of the bubble rupture. The next two cases examine how the volume fraction experiencing damaging stress levels changes when the viscosity or surface tension of the liquid is altered, which is often the case when the cells are grown in different medias. In the first of these two cases, we increase the viscosity of the liquid from 1 mPa s to 1.58 mPa s while holding all other liquid properties and the bubble size constant. This increase in viscosity is equivalent to reducing the Laplace number from the first case with water of La = 3.5 × 10^4^ to La = 1.4 × 10^4^. Next, to determine the volume fraction experiencing damaging EDR levels, we calculate the dimensionless EDR by dividing the chosen threshold by the characteristic EDR (10^7^ W m^−3^)/EDR_c_ = 3.66 × 10^−9^, which is approximately equal to the bottom curve (red triangles). This combination of dimensionless Laplace and EDR numbers corresponds to an effected volume percentage of 4.4% (Fig. 6b, point B). Finally, we evaluate how the addition of a particular surfactant, which reduces surface tension from 72 mN m^−1^ to 40.5 mN m^−1^, would alter the volume of the liquid affected by the specified EDR. Again, we calculate both the dimensionless EDR value 3.66 × 10^−9^ and the Laplace number La = 2 × 10^4^, which corresponds approximately to point C in Fig. 6b. In contrast with the case of increased viscosity, there is a significant reduction in the percentage from 4.6% for water to 1.9% when the surface tension—the driving force of the bubble rupture—has been reduced. Thus, these cases illustrate how altering the viscosity and surface tension of the liquid can modify the number of cells that might be damaged from the bubble rupture.

## Conclusions

This paper quantifies the potential for bursting bubbles to damage cells below the free surface. In particular, our results highlight the concerns of relying on a single temporal and spatial maximum energy dissipation rate when determining the potential for a bursting bubble to damage surrounding cells. Here, we report maximum EDRs exceeding 10^15^ W m^−3^, orders of magnitude higher than previous estimates. However, because of the finite-time singularity associated with this particular flow, the maximum EDR is unbounded and therefore computed values will increase with increasing mesh resolution. Yet, the volume over which this EDR_max_ acts decreases with increasing mesh resolution, diminishing its overall impact. Therefore, we find that reporting a single maximum EDR for the entire bubble rupturing process is problematic. Instead, we propose reporting the liquid volume over which a chosen EDR threshold has been exceeded, which our results demonstrate is far less sensitive to mesh resolution. We expect cells contained within this calculated volume to be damaged if their resilience is below this chosen threshold.

Our results indicate that the volume of liquid experiencing an elevated level of EDR increases with bubble size for a given set of liquid properties. However, because smaller bubbles damage a larger relative volume, at a fixed sparging flow rate the smaller bubbles would be expected to damage a larger number of cells. Our results also indicate how the liquid properties can influence the volume exceeding a stress that has been linked to cell damage, which can be evaluated using a dimensionless framework. We anticipate that our results will be helpful in quantifying the impact of a chosen bubble distribution for aeration on cell viability and lead to a more holistic approach in bioreactor modeling and design. Beyond the biotechnology industry, we believe the results of this work may also apply to the optimization of other fermentation processes such as biofuel production and commercial brewing. On an even larger scale, the EDRs predicted in our study might shed light into the levels of stress imparted to microorganisms in the upper-most layer of the ocean following the rupture of bubbles in a white-cap.
